# Cognitive dual-task cost depends on the complexity of the cognitive task, but not on age and disease

**DOI:** 10.3389/fneur.2022.964207

**Published:** 2022-10-03

**Authors:** Edoardo Bianchini, Elke Warmerdam, Robbin Romijnders, Clint Hansen, Francesco E. Pontieri, Walter Maetzler

**Affiliations:** ^1^Department of Neurology, Kiel University, Kiel, Germany; ^2^Department of Neuroscience, Mental Health and Sensory Organs (NESMOS), Sapienza University of Rome, Rome, Italy; ^3^Division of Surgery, Saarland University, Homburg, Germany; ^4^Faculty of Engineering, Kiel University, Kiel, Germany; ^5^Santa Lucia Foundation, Istituto di Ricovero e Cura a Carattere Scientifico (IRCCS), Rome, Italy

**Keywords:** dual task cost, Parkinson's disease, stroke, Multiple Sclerosis, low back pain, Stroop test, reaction time, dual task

## Abstract

**Introduction:**

Dual-tasking (DT) while walking is common in daily life and can affect both gait and cognitive performance depending on age, attention prioritization, task complexity and medical condition. The aim of the present study was to investigate the effects of DT on cognitive DT cost (DTC) (i) in a dataset including participants of different age groups, with different neurological disorders and chronic low-back pain (cLBP) (ii) at different levels of cognitive task complexity, and (iii) in the context of a setting relevant to daily life, such as combined straight walking and turning.

**Materials and methods:**

Ninety-one participants including healthy younger and older participants and patients with Parkinson's disease, Multiple Sclerosis, Stroke and cLBP performed a simple reaction time (SRT) task and three numerical Stroop tasks under the conditions congruent (StC), neutral (StN) and incongruent (StI). The tasks were performed both standing (single task, ST) and walking (DT), and DTC was calculated. Mixed ANOVAs were used to determine the effect of group and task complexity on cognitive DTC.

**Results:**

A longer response time in DT than in ST was observed during SRT. However, the response time was shorter in DT during StI. DTC decreased with increasing complexity of the cognitive task. There was no significant effect of age and group on cognitive DTC.

**Conclusion:**

Our results suggest that regardless of age and disease group, simple cognitive tasks show the largest and most stable cognitive effects during DT. This may be relevant to the design of future observational studies, clinical trials and for clinical routine.

## Introduction

In daily life, it is very common to encounter situations that require performing two tasks simultaneously, such as walking and talking on the phone. In research conditions, this is referred to as dual-task (DT), as opposed to single-task (ST). In particular, a large body of research has focused on the interaction between cognitive tasks (e.g., serial subtractions) and motor tasks (e.g., walking), showing that DT can affect the performance of one or even both of the tasks involved compared to ST (1). The effect of DT can be calculated as the percentage change in the parameters on interest between DT and ST and has been termed DT cost (DTC). A positive value of DTC means that performance worsens in DT compared with ST and vice versa (1). In motor-cognitive DT paradigms, DTC can be calculated for both cognitive and motor tasks, reflecting an improvement or decrease in either motor or cognitive performance or both. The DTC has been widely used to study the interaction between movement and cognition as a reliable measure of the effect of DT in both physiological (2) and pathological conditions (3–5). In addition, it has been shown to be a useful outcome measure for assessing response to training programs and rehabilitation interventions (6–10). Indeed, a reduction in DTC reflects better motor-cognitive integration and attentional capacity (1).

Walking is one of the most common motor activities performed daily, and a large number of situations involving DT occur during walking. In addition, factors that negatively affect the ability to walk, such as neurological disorders, orthopedic impairments, and balance disorders, have a profound impact on people's independence and quality of life (11). Several studies have reported deterioration in gait parameters, such as reduced gait speed (4, 12–14), cadence (4, 12) and increased gait variability (4, 12, 15, 16), in DT compared with ST. These effects can be observed in different age groups such as healthy older (12) and younger adults (16), and disease populations such as Parkinson's Disease (PD) (14), Multiple Sclerosis (MS) (13), stroke (4) and chronic low-back pain (cLBP) patients (15). Several mechanisms have been proposed to explain the effect of DT, such as attention allocation theory and the bottleneck theory (1). The attention allocation theory states that people have a limited attention capacity. When, as in multitasking, attention demands exceed this capacity, performance on one or more of the simultaneously performed tasks decreases. The bottleneck theory states that above a certain threshold of processing load, only one task can be completed at a time due to limited cognitive resources, resulting in a decline in performance (1). However, many studies have reported no relevant or even negative cognitive DTC, both in healthy participants (17–23) and in patients with cLBP (15) and neurological conditions (24–31). Based on these observations, Plummer and colleagues proposed a classification of the motor-cognitive interactions of DT into 9 possible outcomes based on the combination of effects (i.e., improvement, no change or worsening compared with ST) in cognitive and motor performance, respectively (32). The variability in the results reflects the complexity of interactions between motor and cognitive tasks, and several explanations have been proposed. One possible explanation is that the two tasks do not compete for the same resource pool, resulting in little or no interference between the two. Another hypothesis suggests that a cross-talk interaction might exist and that the two tasks interact in a facilitative manner when they derive from similar cognitive domains and use similar neural populations (1, 33).

Another factor involved in the interaction between gait and cognitive tasks is task complexity, but only a few studies have investigated its influence on cognitive DTC, with variable results. Some studies have reported higher cognitive DTC as cognitive task complexity increases (34–36). The effect seems to be influenced by age and cognitive domains involved (35–39). However, another study did not find this effect (40).

Requiring demanding attentional skills and the integrity of executive functions (1), DT has been used to study cognitive and motor interaction in various physiological and pathological conditions, such as aging, neurological disorders and cLBP. It has been reported that older participants have a reduced ability to process multiple tasks simultaneously, resulting in a higher cognitive DTC compared with younger adults (2) and this is probably related to reduced processing speed and cognitive reserve (37, 41, 42). Some studies have also investigated the effect of motor-cognitive DT on cognitive DTC in various neurological disorders such as PD (43), MS (6, 44) and stroke (4). However, these studies have focused only on single neurologic conditions or age groups.

Recently, cLBP has been shown to be associated with impaired attention and working memory (45) and altered connectivity of the attentional network (46), and DT have led to impairment of gait and balance parameters in these patients (15, 47, 48). Only one study has reported data on cognitive DTC in patients with cLBP, showing little or no effect of DT on verbal fluency (15). However, it is unclear whether more challenging concomitant cognitive tasks can unmask an effect of DT on cognitive performance. Based on clinical experience, we hypothesized that this cohort might show low DTC or even an improvement in cognitive performance in DT with more challenging cognitive tasks due to a pain distraction effect.

Therefore, the aim of the present study was to investigate the effects of DT on cognitive DTC (i) in a dataset including participants of different age groups, with different neurological conditions and cLBP, (ii) at different levels of cognitive task complexity, and (iii) in the context of a setting relevant to daily life, such as combined straight walking and turning. The idea for the experimental paradigm was based on the fact that short walking episodes combined with turns better reflect everyday situations than the straight walking paradigms used in previous studies (49) and that we assumed that such combined straight walking and turning movements are often performed under DT conditions in daily life.

## Methods

In the present study we analyzed a dataset from a cross-sectional study designed to develop and validate mobility algorithms. This included participants with different age groups, with different neurological disorders and cLBP. Detail of the dataset protocol can be found in (50).

### Population

Participants were longitudinally recruited through flyers placed in public facilities (healthy participants) and in neurology departments and outpatient clinics at University Hospital Schleswig-Holstein (UKSH), Kiel Campus, Germany (neurological patients). Inclusion criteria were (i) age 18 years or older and (ii) ability to walk independently without walking aids. Exclusion criteria were (i) Montreal Cognitive Assessment score <15 and (ii) other movement disorders affecting mobility performance, as judged by the assessor. Participants were divided into 6 groups according to age and diagnosis. Healthy participants were divided into “younger” (age 18–45 years) and “older” (age ≥ 60 years). Participants with neurological disorders included patients with PD [according to the UK Brain Bank criteria (51)], MS [according to McDonalds criteria (52)], recent symptomatic stroke (<4 weeks), and cLBP, diagnosed on the basis of the patient's medical history and examination (53).

### Ethics

The study was approved by the ethical committee of the Medical Faculty of Kiel University (D438/18) and was conducted in accordance with the principles of the Declaration of Helsinki. All participants provided written informed consent before the start of measurements. The study is registered in the German Clinical Trials Register (DRKS00022998).

### Demographic and clinical data

Demographic data including age, sex, weight, and height were collected. Overall cognitive function was assessed with the Montreal Cognitive Assessment (54). Mobility was assessed with the Short Physical Performance Battery (SPPB) (55). Disease-specific evaluations included the MDS Unified Parkinson's Disease Rating Scale part III (MDS-UPDRS-III) (56) and the Hoehn and Yahr Scale (57) for PD patients; the Expanded Disability Status Scale (58) for MS patients; the NIH Stroke Scale (59) for patients with stroke; a visual analog scale of pain (60) and the German Funktionsfragenbogen Hannover Scale (61) for patients with cLBP.

### Experimental procedure

Participants performed two smartphone-based cognitive tasks of different complexity. The first task consisted of a simple reaction time test (SRT) in which participants had to tap the screen as fast as possible after the appearance of a black square. Six time intervals ranging from 1,000 to 2,000 ms (in 200 ms increments) were used for the appearance of the black square. Each condition appeared 4 times for a total of 24 trials, administered in random order. The response time in ms was recorded for each trial, and the average of all trials was calculated for each participant. The second task consisted of a numerical Stroop test in which participants had to choose the higher number from two options (62). The Stroop test was administered in 3 conditions: (i) Congruent, in which the number with a higher value had a larger character size (StC); (ii) Neutral, in which the character size of both numbers was equal (StN); (iii) Incongruent, in which the number with a higher value had a smaller character size (StI). Each condition consisted of 8 trials for a total of 24 total trials in random order. The SRT and Stroop tasks were performed consecutively. Response times were recorded in ms and the average for each condition was calculated for each participant. Both the SRT and Stroop numerical tests were performed on a smartphone while standing quietly (ST condition) and while walking up and down a 5-meter walkway (DT condition). For the DT condition, participants were not given any instruction regarding task prioritization.

### Movement and rotation analysis

Participants were measured with a 3D optical motion capture system (Qualisys AB, Gothenburg, Sweden). Reflective markers were placed on the head, sternum, pelvis, and feet. The exact placement of the markers is described elsewhere (50). Data were recorded at 200 Hz. To detect turns during walking, pelvis angles were calculated according to Euler's method. The beginning and end of a turn were detected by the change in the standard deviation of the angular signal around the vertical axis, using the Matlab's built-in function *findchangepts*.

### Data analyses

Turning phases were extracted by the method described above, the total time spent during turning was computed and used to calculate the turning ratio according to the following formula:


$$\begin{array}{l} Turning\;ratio\;=\;\frac{Turning\;time}{Total\;trial\;time}\times 100 \end{array}$$

To assess the impact of DT on cognitive performance, DTC was calculated using the following formula (63, 64):


$$\begin{array}{l} DTC=\frac{Dual\;task-Single\;task}{Single\;task}\times 100\; \end{array}$$

### Statistical analysis

Statistical analyses were performed using JASP v0.16.1 (JASP Team, University of Amsterdam), R v4.0.3 and RStudio v2022.02.2+433 for Windows (R Foundation for Statistical Computing, Vienna, Austria). Descriptive statistics were calculated for the examined variables. To assess the difference in DTC across the 6 groups and different cognitive tasks, a mixed ANOVA test was used, with the factors “task” and “group.” To assess the difference in response times across the 6 groups between ST and DT, separate mixed ANOVA tests were used, one for each cognitive task, with the factors “condition” and “group.” *Post-hoc t*-tests with Bonferroni's correction for multiple tests were performed in case of significant ANOVA main effects. If necessary, Greenhouse-Geisser's correction for non-sphericity were applied. Partial eta squared (${\text{η}}_{\text{p}}^{2}$) were reported as measure of effect size. The significance threshold was set at α <0.05. All data were reported as mean ± SD or median (Q1–Q3) for numerical data and N (%) for categorical variables.

## Results

A total of 91 participants were enrolled in the study. Table 1 shows the demographic and clinical data of the included population. The average turning ratio was 23%.

**Table 1:** Demographics and clinical scores of the enrolled participants.

		**Younger (*N* = 26)**	**Older (*N* = 16)**	**PD (*n* = 19)**	**Stroke (*N* = 9)**	**MS (*N* = 16)**	**cLBP (*N* = 5)**
Age		28.2 ± 8.8	72.3 ± 6.4	63.5 ± 11.8	62.4 ± 19.2	38.1 ± 12.9	62.8 ± 19.8
Sex	M	11 (42%)	8 (50%)	7 (37%)	2 (22%)	10 (62%)	2 (40%)
	F	15 (58%)	8 (50%)	12 (63%)	7 (78%)	6 (38%)	3 (60%)
Height		180.6 ± 8.7	172.9 ± 9.4	174.8 ± 9.0	177.3 ± 10.5 + 8	177.3 ± 11.6	174.0 ± 8.8
Weight		75.1 ± 14.5	79.1 ± 17.1	81.8 ± 16.4	79.4 ± 15.9	77.4 ± 11.2	76.6 ± 11.9
BMI		22.9 ± 3.0	26.4 ± 5.1	26.6 ± 4.1	25.1 ± 3.9	24.7 ± 3.7	25.2 ± 2.6
MoCA		29 (28–29.75)	22.5 (21–27.25)	25 (23–26.5)	24 (22.5–26.25)	28 (26–28.25)	25 (24–27)
SPPB		12 (12–12)	11 (9–11)	10 (8–11)	11 (11–11)	9 (7–11)	12 (7–12)
H&Y		-	-	2 (1–3)	-	-	-
MDS-UPDRS-III		-	-	20 (12–28)	-	-	-
EDSS		-	-	-	-	1 (1–3.5)	-
NIHSS		-	-	-	0 (0–1)	-	
pVAS							3.5 (1.5–5.75)
FFbH							30 (29–31)

MoCA, Montreal Cognitive Assessment; SPPB, Short Physical Performance Battery; H&Y, Hohen and Yahr scale; MDS-UPDRS-III, MDS Unified Parkinson's Disease Rating Scale part III; EDSS, Expanded Disability Status Scale; NIHSS, NIH Stroke Scale; pVAS, Pain Visual Analog Scale; FFbH, Funktionsfragenbogen Hannover. Values are reported as Mean ± SD or Median (Q1–Q3) for numerical variables and N (%) for categorical variables.

### Effect of complexity of the cognitive tasks

**Figure 1:**
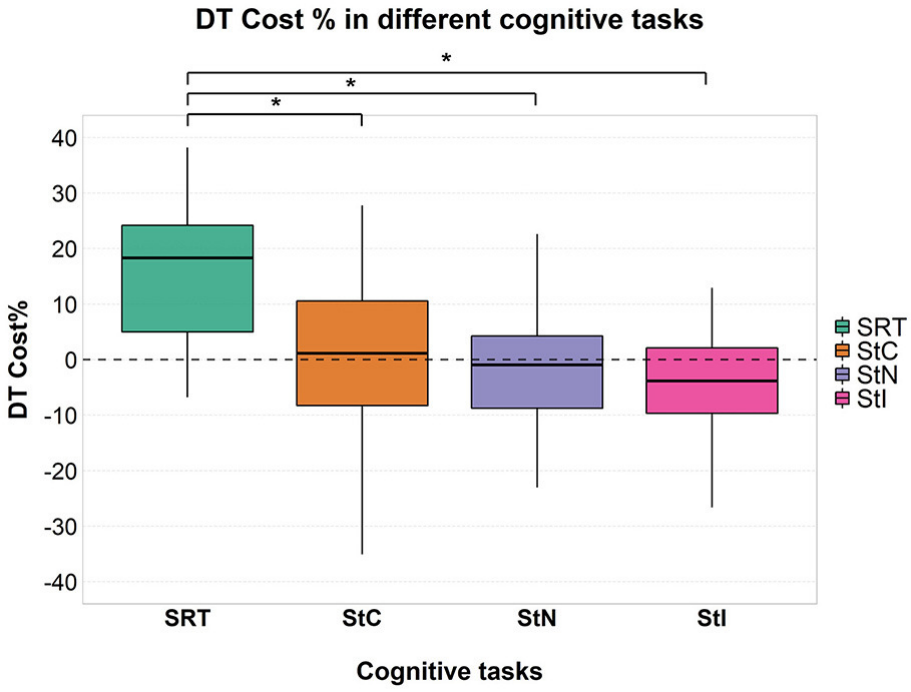
Cognitive DTC in each cognitive task. A negative value means a reduced response time and a consequently better performance and vice versa. A dashed line to mark the zero was added. Significant pairwise comparisons are marked with an asterisk. SRT, Simple Reaction Time; StC, Congruent Stroop Test; StN, Neutral Stroop Test; StI, Incongruent Stroop Test.

**Figure 2:**
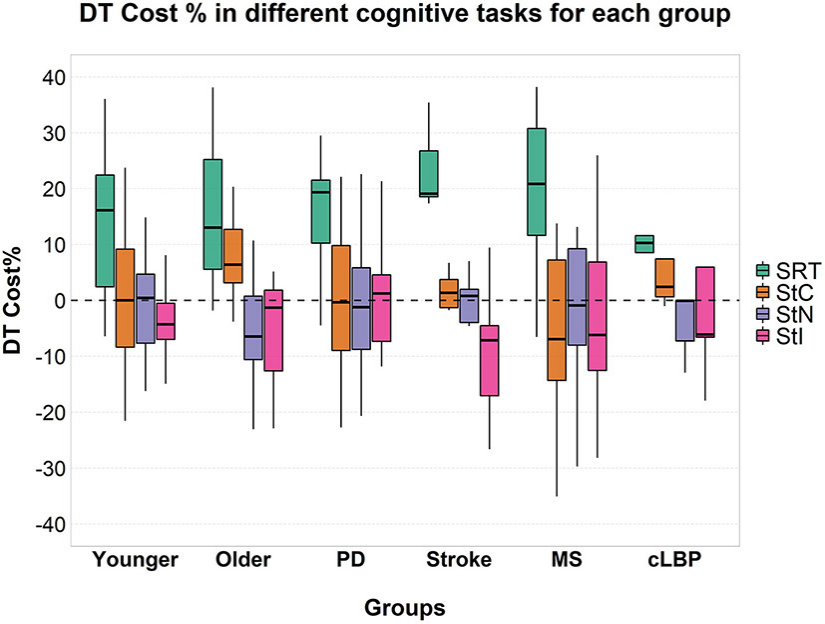
Cognitive DTC across the different cognitive tasks in each group. A negative value means a reduced response time and a consequently better performance and vice versa. A dashed line to mark the zero was added. SRT, Simple Reaction Time; StC, Congruent Stroop Test; StN, Neutral Stroop Test; StI, Incongruent Stroop Test.

Mixed ANOVA showed a significant effect of factor “task” [F_(3, 177)_ = 48.630; *p* < 0.001; ${\text{η}}_{\text{p}}^{2}$ = 0.452] (Figure 1) and *post-hoc* analysis showed significantly higher cognitive DTC in SRT than in all Stroop conditions (all *p* < 0.001), with no significant differences among these in the overall group and in all subgroups except in patients with cLBP (Figure 2).

**Figure 3:**
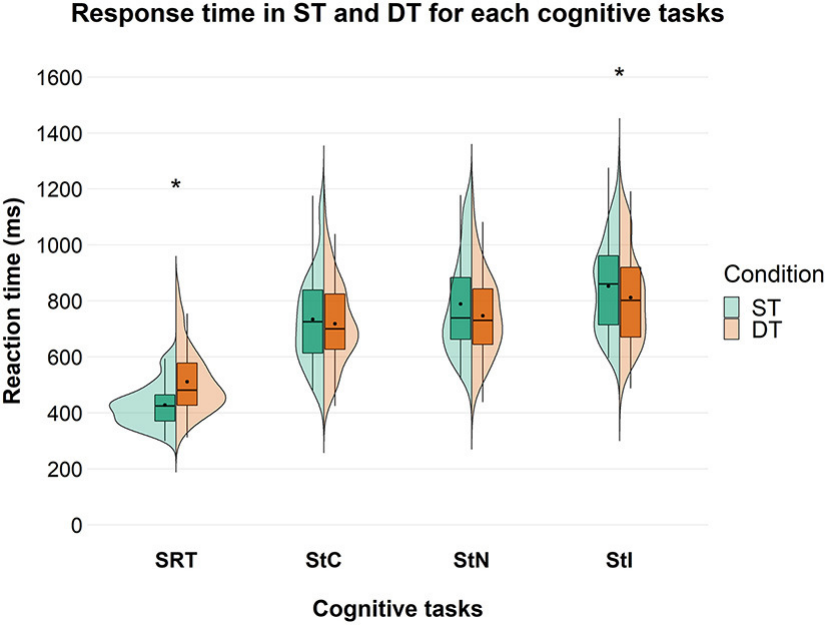
Response time in ms between ST and DT, across the different cognitive tasks in the overall group. Significant differences between ST and DT are marked with an asterisk. SRT, Simple Reaction Time; StC, Congruent Stroop Test; StN, Neutral Stroop Test; StI, Incongruent Stroop Test.

A significant effect of factor “condition” [F_(1, 85)_ = 71.999; *p* < 0.001; ${\text{η}}_{\text{p}}^{2}$ = 0.459] was found on the response time for the SRT task with an overall higher response time in DT than in ST condition. Considering Stroop test, a significant effect of factor “condition” was found only for StI [F_(1, 71)_ = 9.411; *p* = 0.003; ${\text{η}}_{\text{p}}^{2}$ = 0.117] with an overall decrease in response time in DT compared to ST condition. *Post-hoc* analysis showed that response time was significantly higher in DT than in ST in all groups in SRT. On the other hand, in StI, response time was significantly lower in DT than in ST only in the younger participants, while no significant difference was found in the other groups (Figure 3).

Cognitive DTC values for all groups in each cognitive task are shown in Supplementary Table 1.

### Effect of age and disease group

**Figure 4:**
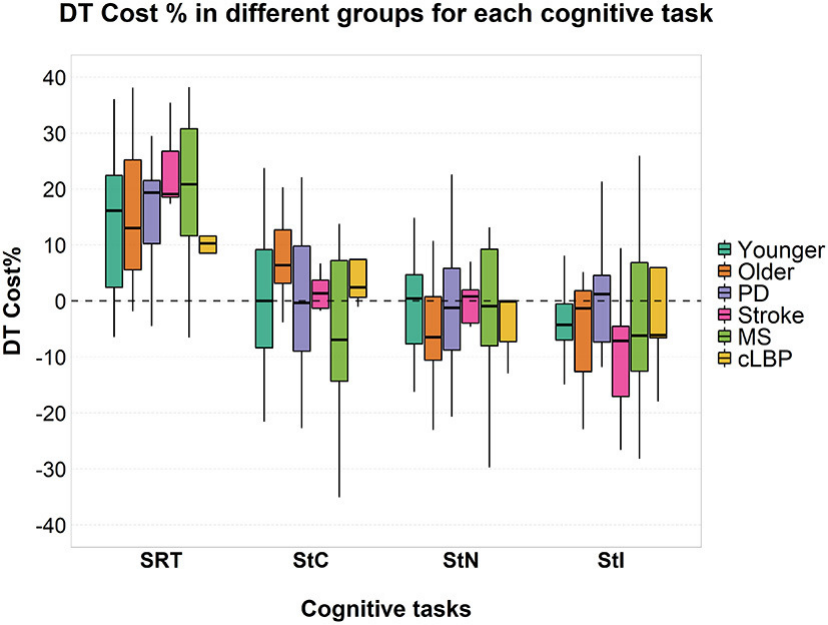
Cognitive DTC across the different groups in each cognitive task. A negative value means a reduced response time and a consequently better performance and vice versa. A dashed line to mark the zero was added. SRT, Simple Reaction Time; StC, Congruent Stroop Test; StN, Neutral Stroop Test; StI, Incongruent Stroop Test.

No significant effects of factor “group” or interaction between the two factors were found on cognitive DTC (Figure 4).

A significant effect of factor “group” was found [F_(5, 85)_ = 8.000; *p* < 0.001; ${\text{η}}_{\text{p}}^{2}$ = 0.320] on the response time for the SRT task and a significant interaction between factor “condition” and “group” [F_(5, 85)_ = 3.022; *p* = 0.015; ${\text{η}}_{\text{p}}^{2}$ = 0.151]. A significant effect of factor “group” was found for StC [F_(5, 70)_ = 10.626; *p* < 0.001; ${\text{η}}_{\text{p}}^{2}$ = 0.432], StN [F_(5, 68)_ = 10.663; *p* < 0.001; ${\text{η}}_{\text{p}}^{2}$ = 0.439] and StI [F_(5, 71)_ = 15.337; *p* < 0.001; ${\text{η}}_{\text{p}}^{2}$ = 0.519]. No significant interaction was found between the two factors for all three conditions of Stroop test (Figure 4). *Post-hoc* pairwise comparisons for factor “group” and details of response time in ST and DT are shown in detail in Supplementary Table 2.

## Discussion

This analysis on a cross-sectional dataset aimed to evaluate the effect of motor-cognitive DT at different cognitive task complexities on cognitive DTC during turning and walking in healthy participants, patients with different neurological conditions, and patients with cLBP. We found a significant effect of cognitive task complexity on cognitive DTC while age and disease did not have a significant effect on cognitive DTC in the population studied here. These results are discussed in detail in the following.

### Effect of complexity of the cognitive tasks

Cognitive DTC depended on cognitive task complexity, both in the overall group and at the subgroup level, with DTC decreasing as task complexity increased. This is in contrast to what we expected. A significant difference in response time between ST and DT was found during SRT and StI, but not during StC and StN. During SRT, a higher response time was observed in DT, compared with ST (Figure 3) and this can be seen in all subgroups (Supplementary Table 2). In contrast, during StI, we observed a lower response time in DT, compared to ST (Figure 3). Our results on SRT are in line with previous studies showing a deterioration in reaction time when performing another task simultaneously, such as contralateral movements (65), a secondary cognitive task (66), a postural task (67) or walking (68, 69). However, previous studies have reported that cognitive DTC is highly variable depending on several factors such as attention (70), age and gender (18, 35, 36, 38, 71–74), task type and difficulty (35, 36, 39, 63, 70, 75), and these variables show a complex interaction. In addition, several studies have shown that cognitive performance in DT did not change compared to ST (17, 19–21) or even improved (17, 18, 20, 29). This has been demonstrated in both healthy participants and patients with neurological conditions and cLBP (15, 24–28, 30, 31). The opposite results between SRT and StI, found in this study, could be due to several mechanisms, such as (i) attention prioritization; (ii) the different cognitive functions involved in the two tasks; and (iii) the interaction between the learning processes within sessions and task complexity.

Attention and task prioritization have been shown to have an important effect on motor-cognitive DT involving walking (18, 24, 64, 67, 76). In our study, the task context was similar for each condition, but participants were not instructed to prioritize the cognitive task or walking and turning, thus they may have adopted a “cognitive first” strategy in StI and a “mobility first” strategy in SRT. A similar strategy adaptation has already been described for older adults (77). In accordance with our results, previous studies have also suggested a role of task complexity in the choice of different attentional strategies, with more complex cognitive tasks resulting in lower DTC and simpler cognitive tasks resulting in higher DTC (78).

SRT and Stroop tasks require different cognitive functions. The SRT is a measure of processing speed (79), while the Stroop test is a more complex task involving executive functions (80, 81). Previous studies have demonstrated an important role of executive functions in gait, and their impairment has been associated with reduced performance in walking tasks (82). According to the cross-talk model (1), the greater involvement of executive functions in both StI and walking could explain the lower DTC during this condition. In contrast, during SRT, the different cognitive functions involved might have led to a negative effect and a higher DTC with a significant difference compared to all Stroop conditions, both in the overall group (Figure 1) and at a the subgroup level (Figure 2).

As SRT is a simple task, it is also possible that the learning curve was so fast that DT effects were well-presented with this paradigm, but that the learning curve was substantially lower for the Stroop tasks which may have led to better results during the DT sessions (which were always performed after the ST sessions).

### Effect of age and disease group

Differently from what we expected, we found no significant differences across groups in cognitive DTC (Figure 4). This result suggests that neurologic diseases and cLBP, at the level of severity and age included in the present study, might have no relevant effect on cognitive DTC. Previous findings have shown higher cognitive DTC in older adults compared with younger ones (2). This has been linked to reduced cognitive reserve and slowed processing capacity, with some cognitive domains (e.g., executive functions) more involved than others (37, 41, 42). The greater cognitive demands of walking in older participants may also play a role in these observations. Indeed, at a younger age, walking is a task with a high level of automaticity, whereas this decreases with age, leading to more active control and, consequently, a higher cognitive load (42). This is also demonstrated by the higher level of activation of the prefrontal cortex during walking with increasing age (83). Previous studies also reported higher cognitive DTC in PD (43, 84) and stroke patients (4) compared with healthy participants. This has been linked to physical and cognitive impairment related to these disorders and to reduced attention and cognitive reserve (85). In MS patients, on the other hand, studies have shown a negative effect of DT on cognitive performance (6, 86), but only a few studies have reported data on cognitive DTC and it remains unclear whether this differs between MS patients and healthy participants (3). Finally, only one study reported data on cognitive DTC in patients with cLBP showing little or no effect of DT on cognitive performance (15).

Finally, we found no interaction between the factor “complexity” and “group” on cognitive DTC. Previous studies have reported an influence of age on the effect of task complexity on DTC, with younger participants showing a significant DTC on cognitive performance only in the most challenging cognitive tasks, whereas older participants were reported to be more prone to DT effect at a lower level of cognitive task complexity (37). The domain-specific effect of DT on cognitive performance also appears to differ between younger and older adults (38, 39). The discrepancies between the previous results and ours might lie in the low disease severity of our patients and their overall good cognitive and physical function. Therefore, we might hypothesize that at the disease severity included in our study, the specific features of the disorders in terms of physical and cognitive impairment have little impact in cognitive DTC. However, most studies have focused on single conditions or age groups and, to our knowledge, there has been no study in which participants with different neurological conditions, cLBP and of different ages have been included in the same experimental protocol.

### Limitations

We acknowledge that this study has limitations. First, the sample size of some groups, in particular the cLBP group, is low. This is explained by the basic design of the cross-sectional study, which provided the dataset used in the present article. However, we believe it is still useful to show data on the entire spectrum of diseases studied to open the possibility of generating new hypotheses for future studies. Second, participants had, on average, relatively high levels of physical and cognitive abilities. This may limit the generalizability of our results, and further studies including participants with lower functional scores may be needed. Third, in DT, participants were not instructed to prioritize any of the tasks. This could have led to different attentional strategies. However, we argue that this problem is difficult to avoid. For example, we cannot exclude that the instruction “Please do not prioritize any of the tasks” does not lead to the activation of another (cognitive) task during the experiment. Fourth, standing while performing a cognitive task might be considered an (additional) resource-consuming task. Indeed, this paradigm has been used in previous studies as a DT postural paradigm (87–89). In future studies, the possibility of performing cognitive tasks while sitting could be considered. Fifth, although none of the participants reported attentional deficit as a specific symptom, we did not assess this aspect in a standardized and structured way, thus an effect cannot theoretically be ruled out. Sixth, we focused our analysis on disease and age groups therefore we did not test the independent effect of age across neurological diseases and cLBP. However, we do not expect this to have substantially affected our data, as we consistently compared disease groups with both older and younger adults in our calculations. Similarly, we focused on the differences across groups without assessing the independent effect of disease severity or functional scores. Future studies will be useful to address this issue. Finally, ST and DT, as well as SRT and Stroop conditions, were performed consecutively. Thus, we cannot rule out that at least some improvement between ST and DT may be explained by a learning effect.

## Conclusion

DT while walking has a different effect on cognition depending on the complexity of the cognitive task. Cognitive tasks that are more complex and involve executive functions showed lower cognitive DTC and even better cognitive performance in DT than in ST. The underlying mechanisms could involve task prioritization, cross-talk interaction, or a combination of learning processes and interference between the two tasks. Age and disease group did not have a relevant effect on cognitive DTC in our cohort. These findings could help movement disorder specialists, neurologists, general practitioners, allied health therapists and patients to interpret the interaction between cognitive abilities and walking in a context that more closely reflects everyday walking conditions, and could be useful to industry and academic principal investigators to design effective assessment batteries that focus on DTC.

## Data availability statement

The raw data supporting the conclusions of this article will be made available by the authors, without undue reservation.

## Ethics statement

The studies involving human participants were reviewed and approved by Ethical Committee of the Medical Faculty of Kiel University (D438/18). The patients/participants provided their written informed consent to participate in this study.

## Author contributions

EW, CH, and WM designed the study. EW collected data. EW and EB performed data analyses. EB and WM wrote the first draft of the manuscript. EW, RR, CH, and FP reviewed manuscript draft. All authors contributed to the article and approved the submitted version.

## Funding

This study was funded by the Open Access Publication fund of the Christian-Albrechts-University of Kiel for scientists of the CAU by Schleswig-Holstein state government. We acknowledge the financial support by DFG within the funding programme Open Access-Publikationskosten.

## Conflict of interest

The authors declare that the research was conducted in the absence of any commercial or financial relationships that could be construed as a potential conflict of interest.

## Publisher's note

All claims expressed in this article are solely those of the authors and do not necessarily represent those of their affiliated organizations, or those of the publisher, the editors and the reviewers. Any product that may be evaluated in this article, or claim that may be made by its manufacturer, is not guaranteed or endorsed by the publisher.
