# Supplementary material for: Cognitive dual-task cost depends on the complexity of the cognitive task, but not on age and disease
Source: Front Neurol. 2022 Oct 3;13:964207. doi: 10.3389/fneur.2022.964207 (PMC9615561; doi:10.3389/fneur.2022.964207)
Supplement: Supplementary file 1 [file Table_1.DOCX]

**Supplementary Table 1.** **Cognitive DTC across the different groups and cognitive tasks.** A negative value means a reduced response time and a consequently better performance and vice versa. SRT: Simple Reaction Time; StC: Congruent Stroop Test; StN: Neutral Stroop Test; StI: Incongruent Stroop Test.

|  | Younger | Older | PD | Stroke | MS | cLBP | Overall |
| --- | --- | --- | --- | --- | --- | --- | --- |
| SRT | 12.6 ± 12.6 | 17.7 ± 14.6 | 18.4 ± 14.1 | 26.0 ± 10.3 | 19.7 ± 14.9 | 9.7 ± 6.6 | 20.1 ± 20.8 |
| StC | 0.7 ± 11.3 | 6.4 ± 12.3 | -1.0 ± 13.2 | -1.3 ± 9.7 | -4.9 ± 15.2 | 1.9 ± 15.1 | 0.3 ± 12.8 |
| StN | -1.9 ± 8.7 | 3.9 ± 11.6 | -1.5 ± 13.7 | -1.5 ± 6.9 | -3.3 ± 14.8 | -2.9 ± 10.1 | -2.4 ± 11.1 |
| StI | -5.0 ± 7.7 | -4.7 ± 8.4 | -0.7 ± 8.6 | -9.6 ± 12.0 | -4.0 ± 14.9 | -1.5 ± 8.3 | -3.4 ± 10.5 |

**Supplementary table 2. Response time in ms in ST and DT across the different groups and cognitive tasks.** Data are reported as mean ± SD. Results of *post-hoc* comparisons for factors group and condition are reported. Significant differences between ST and DT are shown in bold. Significant between-group pairwise comparisons for both ST and DT were reported following the legends at the bottom of the table. SRT: Simple Reaction Time; StC: Congruent Stroop Test; StN: Neutral Stroop Test; StI: Incongruent Stroop Test.

|  | Younger | | Older | | PD | | Stroke | | MS | | cLBP | |
| --- | --- | --- | --- | --- | --- | --- | --- | --- | --- | --- | --- | --- |
|  | **ST** | **DT** | **ST** | **DT** | **ST** | **DT** | **ST** | **DT** | **ST** | **DT** | **ST** | **DT** |
| SRT | **383 ± 60** | **420 ± 63** | **456 ± 84 ^a^** | **564 ± 100 ^a^** | **467 ± 71 ^a^** | **569 ± 117 ^a^** | **422 ± 55** | **563 ± 140 ^a^** | **415 ± 61** | **504 ± 79** | **481 ± 77** | **528 ± 95** |
| StC | 599 ± 94 | 601 ± 96 | 747 ± 195 ^a^ | 839 ± 115 ^a^ | 832 ± 173 ^a^ | 810 ± 132 ^a^ | 814 ± 56 ^a^ | 802 ± 87 ^a^ | 717 ± 152 ^a^ | 669 ± 113 ^b c^ | 773 ± 109 | 787 ± 164 |
| StN | 638 ± 86 | 625 ± 88 | 870 ± 187 ^a^ | 862 ± 102 | 867 ± 188 ^a^ | 837 ± 126 | 815 ± 107 ^a^ | 801 ± 99 | 756 ± 140 ^b^ | 726 ± 162 | 826 ± 154 | 797 ± 142 |
| StI | **700 ± 75** | **664 ± 82** | 998 ± 159 ^a^ | 970 ± 147 ^a^ | 931 ± 145 ^a^ | 919 ± 124 ^a^ | 938 ± 110 ^a^ | 842 ± 111 ^a^ | 820 ± 140 ^a b^ | 782 ± 155 ^b^ | 908 ± 146 ^a^ | 893 ± 161 ^a^ |
| a: significant vs younger participants;  b: significant vs older participants;  c: significant vs PD patients;  d: significant vs stroke patients;  e: significant vs MS patients | | | | | | | | | | | | |
